# Toward an optimal quantitative design method integrating user‐centered qualitative attributes

**DOI:** 10.1002/fsn3.1058

**Published:** 2019-05-29

**Authors:** Cyril Bertheaux, Rosario Toscano, Roland Fortunier, Céline Borg

**Affiliations:** ^1^ LTDS UMR5513 ENISE University of Lyon Saint‐Etienne France; ^2^ National School of Mechanics and Aerotechnics (ENSMA) Futuroscope Chasseneuil France; ^3^ Department of Neurology Research Team CMRR Neuropsychology University Hospital Center of Saint Etienne Saint‐Etienne France

**Keywords:** design variables, mathematical sensory model, optimization, sensory analysis, sensory specifications

## Abstract

The determination of product features, which can be seen as design specifications, is a crucial problem that must be carried out upstream to quickly validate the product configuration according to some attributes in relation to the user perception. To this end, the design methods must evolve toward an analysis compatible with various kind of data that can be qualitative or quantitative. In this paper, a new approach is introduced able to take into account various kind of information in order to determine some quantitative design specifications in accordance with the users perception. This is done through a mathematical formulation that exploit different types of data coming from sensory analysis and physical quantities. This mathematical formulation is then used in an optimization procedure that takes into account a preference order over the sensory attributes. The solution of this optimization problem gives thus the best user‐centered specifications that must be used for the conception of the final product.

## 1 INTRODUCTION

The problem of designing new products integrating human perception is not very new and has been explored under various aspects. In the middle of the 20th century, Osgood, Suci, and Tannenbaum (1957), developed the so‐called Semantic Differential Method (SDM) probably the first approach for measuring subjective judgments of peoples Osgood et al. (1957). This approach could then be used to takes into account the perception of the user for a given product.

In 1980, Kano et al., introduced the “Kano model” that is used to study the importance of some product features for the customer satisfaction (Kano, Seraku, & Takahashi, 1984). This approach can thus be employed to create products in accordance with the user preference. Xue, Zeng, and Koehl (2017) developed a fuzzy genetic method to decide the weightings of sensory attributes according to subjects overall preference toward a product under evaluation. Some other others methods have been introduced see for instance (Nagamachi, 2010; Schütte, 2007; Schütte, Eklund, Axelsson, & Nagamachi, 2004) and references therein for a more detailed study.

However, these various methods cannot be easily used to determine directly usable technical or quantitative design specifications reflecting the users preferences. The more suited approach to this end can be accomplished by Kansei Engineering introduced By Nagamachi (1989, 2010). Kansei engineering is a general purpose approach that places the user at the center of the design process and aim to determine some parameters design also called design specifications that are in accordance with the users perception. Due to its generality this approach can be declined in many ways and there exists a vast literature about Kansei like approaches (see Lévy, 2013).

However, this approach is time consuming and require in general a lot of participants. In particular, one of the crucial step in Kansei is to compare the semantic space with the properties space to determine which features of the properties space match the Kansei words of the semantic space. This is an nonobvious task that requires many iterations before converging to a general concensus (Schütte, 2007). Moreover, this kind of approaches are not well suited to determine the best composition of products such as beverage, food, and cosmetic according to a given performance function.

The main objective of this paper was to determine the optimal composition of a product according to a given order over sensory attributes. This kind of problem cannot be solved directly via the classical optimization methods. Indeed any optimization problem can be formulated as follows

(1) $$\begin{array}{c} \mathrm{minimize}\;f_{0}\left(x\right)\\ \mathrm{subject}\;\mathrm{to}\;f_{i}\left(x\right)⩽0,\;i=1,\cdots ,N_{c}\\ \;x\in D=\{x\in R^{n_{x}}:\underline{x}⪯x⪯\overset{¯}{x}\} \end{array}$$

where $f_{0}:R^{n_{x}}\to R$ is the *objective function* (or *cost function*),that is, the function that we want to minimize,^1^ $f_{i}:R^{n_{x}}\to R$, $i=1,\cdots ,N_{c}$ are the *constraint functions*, and the vector $x=(x_{1},\cdots ,x_{n_{x}})$ is the *optimization variable* also called *decision variable* or *design variable*. The set D is such we call the *search domain*, i.e., the set under which the minimization is performed. The vectors $\underline{x}=\left({\underline{x}}_{1},\cdots ,{\underline{x}}_{n_{x}}\right)$ and $\overset{¯}{x}=({\overset{¯}{x}}_{1},\cdots ,{\overset{¯}{x}}_{n_{x}})$ are the bounds of the search domain and the symbol ⪯ means a componentwise inequality. A vector $x_{f}\in D$ is said feasible if it satisfy the $N_{c}$ constraints $f_{i}$; the set of feasible vector is called the *feasible domain*. The solution of the optimization problem 1 is to find x that belong to the feasible domain while minimizing the objective function. Usually, the functions $f_{0}$, $f_{i}$ are determined according to some physical or technical considerations related to the problem at hand. This is in contrast with sensory analysis where the considered product is characterized by sensory descriptors which are qualitative in nature and thus cannot be directly used in the classical formulation 1.

The main contribution of the proposed method is to determine the design parameters (composition) according to a given preference order over the sensory attributes used to characterize the considered product. To this end, a mathematical sensory model is designed and used in an optimization problem. The solution of this optimization problem gives through some sensory specifications the best user‐centered parameters design that has to be used for the conception of the final product.

It must be noted that the proposed method is not focused on sensory evaluation but on how to exploit the sensory evaluation to obtain a standard optimization problem 1 which can be solved by using available algorithms, like for instance the interior point method, genetic algorithms, (GA), and particle swarm optimization (PSO).

## 2 DETAILED DESCRIPTION OF THE PROPOSED OPTIMAL SENSORY DESIGN METHODOLOGY

**Figure 1:**
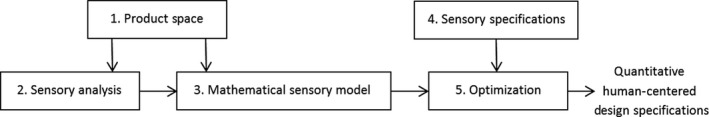
The various steps of the proposed design methodology

Usually, the design of a given product is based on a set of technical specifications that are objective in nature. This means that these specifications are closely related to some physical quantities that have to be satisfied so that the final product accomplishes a given global function with desired performance. However, it can be observed that for products of the same nature, say for instance “razors with four blades,” the technical performances are comparable. Therefore, the act of buying a given product cannot be related only to technical considerations. In fact, it is now well admitted that the act of purchase of a product with respect to another one of similar performance is mainly due to the sensory preferences of the users. In these conditions, the set of specifications that must be done for designing a new product must incorporate not only technical specifications but also sensory specifications in accordance with the users preferences. This is often referred to as the anthropocentric design, i.e., the man must be at the heart of the process design. The main difficulty with the anthropocentric conception is that sensory perception is subjective and therefore not directly measurable as can be a physical quantity. This is the reason why a new discipline appeared, called “sensory evaluation,” where the human being is used as a measurement instrument. The definition of sensory evaluation was first given by the US Food Institute as a: “scientific discipline used to evoke, measure, analyze and interpret reactions to those characteristics of foods and materials as they are perceived by the senses of sight, smell, taste, touch and hearing,” (Gengler, 2009). Nowadays, the application of sensory evaluation is not limited to the food domain and has been extended to any manufactured products in accordance with the to consumers perceptual preference. By this approach, anything that has sensory characteristics perceived by one or more of the human senses can be evaluated, (Gengler, 2009). Figure 1 sketch the proposed design method that takes into account the user perception via the sensory analysis as well as some physical quantities related to the studied product. This approach is based on the solution of an optimization problem that gives the value of some design variables in accordance with a set of a sensory specifications. In the following, each step of the method will be described.

### 2.1 The notion of product space (step 1)

The product space, denoted $P_{K}$, consists of K existing products that correspond, roughly, to the same use functions, but differ in their performance, style or aesthetic etc. Note that, $P_{K}$ is a finite countable set. The products chosen must be different enough to provide a significant variety of stimuli, but close enough to stay in the same sensory domain.

As an example of product space, we can consider 28 samples of a Brazilian beverage with different organoleptic properties, collected from the current market (see Hensen, Pereyra, & Scherer, 2013). Table 1 shows the rough classification of the samples based on their aging time.

**Table 1:** Example of product space

Aging time (in month)	2	3	6	8	12	18	24	36	48	120
Number of samples	1	2	3	2	3	3	7	2	4	1

For these products, some qualitative attributes can be defined and evaluated through a panel of experts. This is done at step 2 of the proposed method (see Figure 1).

### 2.2 Basic principle of the sensory analysis (step 2)

**Figure 2:**
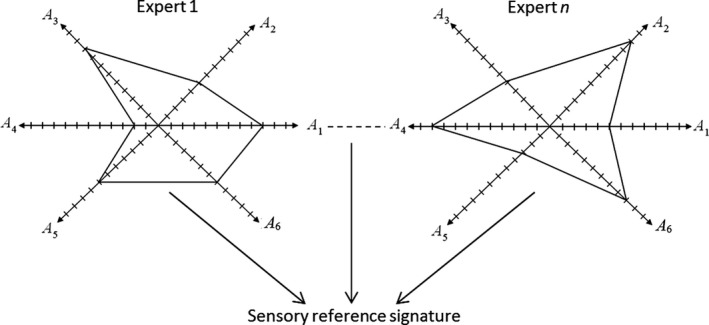
Example of sensory signatures for a given product. Six attributes $A_{1}$ to $A_{6}$ have been defined. The problem is to give a sensory reference signature by aggregating the sensory signature given by each expert. To this end, the mean value aggregation operator can be used

For a given product, various subjective attribute can be defined. For instance, consider a beverage with different organoleptic properties. The attributes can be sweetness, bitterness, length in mouth etc. A panel of experts must be able to evaluate through a scale ranging for instance from 0 to 10 its perception with respect to each attribute. This is the so‐called sensory assessments that must be conducted by a trained panel of experts according to standardized techniques and procedures (see Xue et al., 2017 for a more detailed description). This sensory assessments step gives rise to what is called a sensory signature. Each expert gives its own sensory signature; there is thus one sensory signature per expert. Usually, the mean value is adopted to aggregate the expert advice so that to obtain one and only one sensory signature per product (see Figure 2).

### 2.3 Mathematical sensory model (step 3)

At this stage, we have one sensory reference signature per product of the product space. In addition, for each element of the product space, we have some measurable physical quantities that are chosen so that they are related to the sensory attribute. The problem is to find mathematical relations linking the sensory signature to the measured physical quantities. More formally, we will denote by $S^{\left(j\right)}={\left[A_{1}^{\left(j\right)},\cdots ,A_{m}^{\left(j\right)}\right]}^{T}$, j=1⋯,K, the sensory reference signature of the $j^{\mathrm{th}}$ product of the product space, and by $x^{\left(j\right)}={\left[x_{1}^{\left(j\right)},\cdots ,x_{n}^{\left(j\right)}\right]}^{T}$, j=1⋯,K, the corresponding vector of measured physical quantities. In our notation $A_{i}^{\left(j\right)}$, i=1,⋯,m, represents the $i^{\mathrm{th}}$ sensory attribute of the product number j. Similarly, $x_{i}^{\left(j\right)}$, i=1,⋯,n, represents the $i^{\mathrm{th}}$ measured physical quantity of the product number j. This set of information $\left(S^{\left(j\right)},x^{\left(j\right)}\right)$, j=1,⋯,K, represents the available data base that will be used to define a mathematical sensory model per attribute. Formally, the data base related to the *i*th attribute can be written as follows

(2) $$D_{A_{i}}=\left\{\left(x^{\left(j\right)},A_{i}^{\left(j\right)}\right):x^{\left(j\right)}\in X\subset R^{n},\;A_{i}^{\left(j\right)}\in Y\subset R,\;j=1,\cdots ,K\right\},\;i=1,\cdots ,m$$

where X is the domain of variation of the design variables which are physical quantities, and Y represents the scale used to evaluate each attribute, for instance Y=[0,10].

The problem of sensory modeling is to find a set of mathematical relations of the general form $A_{i}=f_{i}\left(x\right)$, where $f_{i}\left(.\right)$ is an unknown mathematical relation that “explain” $A_{i}$ from the input vector x. Note that, in fact $f_{i}\left(.\right)$ is partially define by the data base $D_{A_{i}}$, i.e., we know only a finite number of input–output data pairs. Since $f_{i}\left(.\right)$ is only partially known, the best we can do is to find a set of empirical models, denoted $g(x,\theta_{i})$, i=1,⋯,m, able to gives an estimate, denoted ${\hat{A}}_{i}$, of the sensory attribute $A_{i}$ for a given x∈X. The empirical model g depends on a parameter vector $\theta_{i}$ that must be tuned so that the estimate ${\hat{A}}_{i}=g\left(x,\theta_{i}\right)$ is as close as possible to the true value $A_{i}$ for all x∈X.

The empirical model is usually built by superposition of some basis functions, we have thus, for each attribute, the following general form

(3) $${\hat{A}}_{i}=g\left(x,\theta_{i}\right)=a_{i,0}+\sum_{k=1}^{p}a_{i,k}g_{k}\left(x,\varphi_{i,k}\right)$$

where $\theta_{i}=\left[a_{i},\phi_{i}\right]$, with $a_{i}=\left[a_{i,0},\cdots ,a_{i,p}\right]$ and $\phi_{i}=\left[\varphi_{i,1},\cdots ,\varphi_{i,p}\right]$. Note that, this model is linear with respect to the parameter vector $a_{i}$ but nonlinear with respect to the parameter vector $\phi_{i}$. This choice has been done to account for the inherent nonlinearity between the physical variables and the sensory attributes. Indeed as observed in (Moskowitz, 2007) the use of a linear model with respect to the parameter vector can lead to poor results when quadratic terms plus eventually cubic terms are introduced.

In our study, the basis functions $g_{k}\left(x,\varphi_{i,k}\right)$, k=1,⋯,p, are chozen as radial basis functions (RBF) of the following form

(4) $$g_{k}\left(x,\varphi_{i,k}\right)=\exp\left(-\frac{\left|\right|\left(x-m_{i,k}\right){\left|\right|}^{2}}{\sigma_{i,k}^{2}}\right),\;k=1,\cdots ,p$$

where $\varphi_{i,k}=\left[m_{i,k},\sigma_{i,k}^{2}\right]$, $m_{i,k}$ is the $k^{\mathrm{th}}$ center of the cluster related to the input space for the *i*th attribute, and $\sigma_{i,k}^{2}$ can be taken as the mean variance within this cluster, i.e., the mean of the diagonal of the covariance matrix. The determination of $m_{i,k}$ and $\sigma_{i,k}$ can be determined via a classical clustering technique like the *k*‐means (Jain, 2010). The optimal number of clusters can be chozen so that the variance within the clusters is as small as possible and so that the smallest distance between the centers is maximal. This criterion can then be used to optimally determine the number of basis functions p.

When this is done, we have to determine the parameter vector $a_{i}=\left[a_{i,0},\cdots ,a_{i,p}\right]$. To this end, note that the set of basis functions $g_{k}\left(x,\varphi_{i,k}\right)$, k=1,⋯,p, define a nonlinear transformation of the input space $X\subset R^{n}$ into another $\mathrm{Xi}\subset R^{p}$. Under this nonlinear transformation, the sensory model 3 is affine with respect to $a_{i}$, indeed, we have

(5) $${\hat{A}}_{i}\left(\xi \right)=a_{i,0}+\sum_{k=1}^{p}a_{i,k}\xi_{k}$$

with $\xi =[\xi_{1},\cdots ,\xi_{p}]\in \mathrm{Xi}$. Thus, the parameter vector $a_{i}$ can be determined via a classical least square regression (see for instance Hensen et al., 2013). The described tuning procedure can be perceived complicated for those not very familiar with function approximation methods. Fortunately, there exists software packages, such as the one described in Beal, Kagan, and Demuth (2018), that can be used for the tuning of the sensory model parameters 3 according to the given data base 2.

### 2.4 Optimization and sensory specifications (steps 4 and 5)

Let $P_{K}$ be the product space of interest. The elements of this space are product of the same nature, i.e., product that are designed to accomplish a given global function. Recall that $P_{K}$ is a finite set that contains K existing products designed to accomplish the same function. This set represents our knowledge base which as been used to determine the relationship between some sensory attributes denoted $A_{i}$, i=1,⋯,m, and a vector of measured physical quantities x∈X. In what follows, x is considered as the design vector that must be determined so that that some sensory specifications are satisfied.

At this step, we have m empirical models (see 2.3) that can be used to estimate the sensory attributes from the design vector x∈X

(6) $${\hat{A}}_{i}\left(x,\theta_{i}\right)=g\left(x,\theta_{i}\right),\;i=1,\cdots ,m$$

Note that given these models, we can generate a continuous space of products. This space denoted P, can be defined as follows

(7) $$P=\left\{{\hat{A}}_{1}\left(x,\theta_{1}\right),\cdots ,\;{\hat{A}}_{m}\left(x,\theta_{m}\right)):\;x\in X,\;A_{i}\left(x,\theta_{i}\right)\in Y,\;i=1,\cdots ,\;m\right\}$$

The space P contain in particular the product that is in good agreement with some sensory specifications. The problem is how to find this “ideal” product? Equivalently, the problem is to find the optimal design vector $x^{*}$ that must be used to generate this ideal product according to the sensory specifications. By sensory specification, we mean a preference order over the sensory attributes. Indeed, some attributes are more important than other to satisfy the the acceptance of the product by the customers. Therefore, these attributes must be ranked by order of preference.

For instance, assume that we have five attributes: $A_{1}$ to $A_{5}$. Suppose that $A_{3}$ and $A_{5}$ have the largest impact on the preference, with $A_{3}$ more important than $A_{5}$, whereas $A_{2}$ has a minor impact and that $A_{1}$ and $A_{4}$ have a negative impact with $A_{1}$ having a less negative impact than $A_{4}$. Then, the preference order is given by the 5‐tuple $\left(A_{3},A_{5},A_{2},A_{1},A_{4}\right)$. To take into account this preference order, we have to maximize those attributes that have the largest positive impact one the preference while respecting the preference order.

Under the preference order specifications, the optimal design vector $x^{*}$ that ensure the maximization of the attributes having the largest positive impact while respecting the preference order is solution to the following optimization problem

(8) $$\begin{array}{c} \mathrm{maximize}\;\sum_{i\in I}w_{i}{\hat{A}}_{i}\left(x,\theta_{i}\right)+\frac{1}{c(x)}\\ \mathrm{subject}\;\mathrm{to}:\;x\in X\\ \;A_{j}\left(x,\theta_{j}\right)>A_{j+1}\left(x,\theta_{j+1}\right),\;j\in J \end{array}$$

where I is the set of indices corresponding to the attributes that have to be maximized, and J represents the set of indices corresponding to the preference order over the attributes. The weight $w_{i}$ reflect the relative importance of the attributes having the largest impact on the preference. These weights are chosen such that they sum to one, i.e., $\sum_{i\in I}w_{i}=1$. For instance, in the case considered before we have I={3,5}, J={3,5,2,1,4}, and $w_{3}>w_{5}$ with $w_{3}+w_{5}=1$.

In the optimization problem 8, c(x) is a positive function related to the financial cost which usually increase with the quality of the components used for realizing the final product. In practice, it is important to take into account the price of the product to be designed. Indeed, a product can be in good agreement with the sensory specifications but too expensive to be attractive. If we want the product accessible for a majority of people, the financial cost must be minimized. The optimization problem 8 can be solved by using available solver such as for instance the Optimization Toolbox described in Coleman, Branch, and Grace (1999).

#### 2.4.1 Determination of the preference order

The order, of preference on the sensory attributes can be determined a priori from market expectations for the product under consideration. Another approach is to use the Xue's method (Xue et al., 2017), in which the preference has been evaluated during the sensory analysis phase by a panel of experts. A fuzzy comprehensive evaluation (FCE) has then be performed to model the relations between the sensory data. The inverse problem of FCE has been solved via a genetic algorithm to determine the weight distribution of the sensory attributes. This weight distribution can then be used in our approach to establish the preference order as well as the determination of the weights associated with the attributes that must be maximized.

The approach adopted in this study consists in conducting a preference test over a large set of users (bigger than 100) of the considered product to obtain a preference score, called hedonic index, for each product of the product space $P_{K}$. A linear regression is then performed between the hedonic score and the normalized sensory data. The coefficient associated with each sensory attribute reflects thus the impact of each attribute to the preference score. The value of these coefficients is then used to determine the weights of the attributes having the largest impact on the preference. This procedure is detailed in the practical example presented in the next section.

### 2.5 Application and limitation of the proposed method

The proposed design method can be used to determine the best composition of beverages, cosmetic products, food products etc., according to some sensory specifications. These specifications are given as a preference order over the sensory attributes used to characterize the product.

The main limitation of the proposed method is that it cannot be used for determining the design parameter of complex products. By complex products, we mean a system such as for instance a car. However, every system can be decomposed in elementary parts which can be eventually if required designed with the proposed method.

Another limitation lie in our ability to solve the nonlinear constrained optimization problem 8. This is particularly true when the size of the design parameter x is large. In this condition, it could be very difficult to find a feasible design vector. To remedy this difficulty, we have to operate a reduction in the design space X. This can be done directly through some physical considerations or via a PCA (Principal Component Analysis). Moreover, we have to keep in mind that only a local maximum can be obtained for this kind of nonconvex optimization problem, but this is sufficient in practice since the preference order is satisfied and the objective function is maximized even locally. If a near global optimum is desired, the problem 8 must be solved many times with a starting point taken at random in the design space.

## 3 PRACTICAL EXAMPLE TO ILLUSTRATE THE PROPOSED DESIGN METHOD

This example is concerned with a Brazilian beverage called “Cachaças”. We have used the sensory data as well as the chemical composition presented in Serafim et al. (2013) to detail the main steps of our method (see Figure 1), notably steps 3, 4, and 5.

### 3.1 Product space and sensory attributes

The product space is composed 28 products (P1 to P28) of the same nature (“Cachaças”) but differ manly in aging time as well as the recipient used to conserve these products.

We have retained 8 sensory attributes related to its taste and aroma, namely: $A_{1}=$ “Burnt,” $A_{2}=$ “Sweetness,” $A_{3}=$ “Bitterness,” $A_{4}=$ “Floral,” $A_{5}=$ “Fruity,” $A_{6}=$ “Vegetable,” $A_{7}=$ “Spicy,” and $A_{8}=$ “Woody.” The sensory attributes were evaluated on a 9‐point scale (“1” for not present to “9” for very much present), by a trained panel (see Serafim et al., 2013 for more details). In addition, an hedonic test was performed by using 240 consumers of this beverage. To this end, a 9‐point scale was used (“1” for “I dislike extremely” to “9” for “I like extremely”). The sensory data and the hedonic score are presented in Tables 2 and 3.

**Table 2:** Sensory signature of products P1 to P14

Product	P1	P2	P3	P4	P5	P6	P7	P8	P9	P10	P11	P12	P13	P14
Hedonic score	5.30	5.30	5.70	5.00	5.80	4.80	5.10	5.20	5.40	5.90	5.40	5.80	5.30	5.10
Sensory attribute *A*_1_	3.25	3.75	4.50	4.17	4.83	4.17	4.67	6.25	4.83	4.92	3.92	3.50	5.58	4.75
Sensory attribute *A*_2_	3.77	3.42	3.92	4.17	4.00	3.17	3.75	4.25	3.92	3.92	3.83	3.75	3.42	3.58
Sensory attribute *A*_3_	2.33	2.67	3.25	3.08	2.75	3.17	4.08	3.17	3.50	3.00	2.75	2.17	3.42	2.67
Sensory attribute *A*_4_	2.42	1.83	1.83	2.67	1.33	2.00	3.00	5.67	4.67	5.42	2.33	2.83	3.25	2.08
Sensory attribute *A*_5_	4.08	4.25	3.92	5.00	4.00	8.83	3.25	5.25	4.91	5.42	4.75	5.33	3.50	4.17
Sensory attribute *A*_6_	2.00	3.00	2.00	2.17	3.67	1.83	3.25	3.42	3.08	1.83	3.08	2.33	2.33	2.50
Sensory attribute *A*_7_	1.75	2.17	1.50	1.83	2.25	2.00	3.33	2.83	3.58	3.58	3.08	2.33	2.50	2.83
Sensory attribute *A*_8_	0.42	0.67	0.83	0.92	3.08	1.08	3.08	2.50	3.75	5.00	3.67	3.75	3.67	4.67

**Table 3:** Sensory signature of products P15 to P28

Product	P15	P16	P17	P18	P19	P20	P21	P22	P23	P24	P25	P26	P27	P28
Hedonic score	5.60	6.40	6.10	6.40	6.50	6.20	6.10	6.30	6.60	6.60	6.20	6.30	6.20	6.00
Sensory attribute *A*_1_	4.17	6.33	4.83	5.25	6.25	6.33	3.83	5.50	3.92	4.83	4.25	3.42	4.92	5.58
Sensory attribute *A*_2_	4.25	3.83	3.50	4.58	4.83	4.08	4.00	4.00	4.58	4.00	4.92	4.33	4.17	4.00
Sensory attribute *A*_3_	2.83	3.33	3.33	2.58	2.08	3.67	2.67	2.58	2.92	3.00	2.67	3.58	2.25	3.17
Sensory attribute *A*_4_	2.25	5.42	5.00	5.00	5.33	5.08	2.83	2.08	3.42	2.08	2.08	3.67	5.42	4.50
Sensory attribute *A*_5_	5.33	5.58	4.83	6.33	5.67	5.25	5.67	5.08	5.58	5.50	4.50	5.75	5.92	5.17
Sensory attribute *A*_6_	2.33	3.00	3.33	2.75	2.67	3.00	2.17	3.17	3.83	2.67	2.75	2.67	2.33	2.92
Sensory attribute *A*_7_	3.50	4.92	4.17	4.92	3.67	4.42	3.00	2.67	3.50	4.58	3.17	5.25	2.92	4.17
Sensory attribute *A*_8_	3.67	5.42	5.08	5.17	5.50	6.58	3.83	4.83	4.92	5.58	3.25	5.92	5.92	5.83

### 3.2 Physical variables

The physical variables correspond here to the chemical composition of each product (see Serafim et al., 2013 for a detailed description). The 16 main chemical ingredients of the product have been considered in this study. The normalized composition, (i.e., between [−1, 1]) of each product is presented in Tables 4 and 5.

**Table 4:** Normalized composition of products P1 to P14

Product	P1	P2	P3	P4	P5	P6	P7	P8	P9	P10	P11	P12	P13	P14
Ingredient 1 (*x*_1_)	−0.796	−0.004	−0.108	−0.106	−1.000	−0.406	−0.163	0.234	−0.607	−0.777	−0.664	−0.822	−0.356	−0.234
Ingredient 2 (*x*_2_)	−0.739	−0.797	−0.857	−0.912	−0.809	−1.000	1.000	−0.879	−0.950	−0.347	−0.815	−0.812	−0.548	0.002
Ingredient 3 (*x*_3_)	−0.784	−0.828	−0.953	−0.973	−0.930	−0.980	−0.277	−1.000	−0.925	−0.492	−0.823	−0.359	−0.722	−0.812
Ingredient 4 (*x*_4_)	−0.301	−0.104	−0.027	−0.956	−0.126	0.628	0.552	0.388	0.224	−0.585	−0.869	−0.934	0.749	−0.060
Ingredient 5 (*x*_5_)	0.587	−0.463	−0.003	−0.473	0.085	−0.302	−0.390	−0.504	−0.158	−1.000	−0.245	−0.494	1.000	0.271
Ingredient 6 (*x*_6_)	−0.214	−0.605	−0.729	−0.722	−0.605	−0.585	−0.717	−1.000	−0.775	−0.722	−0.376	−0.407	−0.315	−0.503
Ingredient 7 (*x*_7_)	−0.839	1.000	−1.000	0.350	−0.695	−0.606	−0.248	−0.516	−0.996	−0.905	−0.970	−0.949	−0.965	−0.952
Ingredient 8 (*x*_8_)	−0.467	0.265	−0.807	−0.563	−0.313	−1.000	0.073	−0.460	−0.560	0.833	0.073	0.007	−0.647	−0.405
Ingredient 9 (*x*_9_)	0.192	0.273	0.313	−1.000	0.192	−0.010	−0.333	0.131	−0.333	−0.354	0.212	−0.333	−0.152	1.000
Ingredient 10 (*x*_10_)	−0.982	−0.837	−0.697	−0.839	−0.815	0.269	−0.781	−0.684	−0.056	1.000	−1.000	−0.656	0.084	−0.982
Ingredient 11 (*x*_11_)	1.000	−1.000	−0.995	−0.982	−0.761	−0.819	−0.757	−0.985	−0.965	−0.773	−0.765	−0.887	−0.909	−0.469
Ingredient 12 (*x*_12_)	−0.669	0.028	−0.934	−0.657	−0.392	−1.000	0.028	−0.481	−0.547	0.370	−0.238	−0.282	−0.746	−0.337
Ingredient 13 (*x*_13_)	−0.951	0.158	0.988	−1.000	−0.948	−0.832	−0.890	−0.890	−0.367	−0.419	−0.983	−0.951	0.318	−0.985
Ingredient 14 (*x*_14_)	1.000	−1.000	−0.173	−0.173	−0.099	−0.200	−0.118	−1.000	−1.000	−1.000	0.417	−1.000	−0.171	−0.140
Ingredient 15 (*x*_15_)	−0.812	−0.899	−0.982	−0.930	−1.000	−0.918	−0.845	−0.959	−0.980	−0.906	−0.545	−0.649	−0.592	−0.927
Ingredient 16 (*x*_16_)	0.250	−0.919	−0.919	−0.919	−0.589	−0.919	1.000	−0.979	−0.679	0.880	−1.000	−1.000	0.010	−0.529

**Table 5:** Normalized composition of products P15 to P28

Product	P15	P16	P17	P18	P19	P20	P21	P22	P23	P24	P25	P26	P27	P28
Ingredient 1 (*x*_1_)	−0.753	0.696	−0.453	0.826	0.913	−0.128	−0.683	−0.612	−0.755	1.000	−0.679	0.371	−0.347	−0.688
Ingredient 2 (*x*_2_)	0.055	−0.326	−0.511	0.271	−0.327	−0.389	−0.576	0.393	−0.115	0.244	−0.759	−0.591	−0.406	0.283
Ingredient 3 (*x*_3_)	0.015	−0.331	−0.692	0.734	1.000	−0.618	−0.274	−0.320	−0.595	0.276	−0.935	−0.692	−0.575	−0.323
Ingredient 4 (*x*_4_)	1.000	0.923	−0.322	0.574	0.956	−0.628	−1.000	0.224	0.137	0.421	−0.902	0.060	−0.180	−0.169
Ingredient 5 (*x*_5_)	−0.736	−0.380	0.209	−0.333	−0.307	−0.204	0.282	−0.307	0.592	−0.621	−0.090	−0.271	−0.318	−0.106
Ingredient 6 (*x*_6_)	−0.483	−0.112	−0.447	0.020	0.436	1.000	−0.102	−0.544	−0.447	0.563	−0.376	−0.173	−0.153	−0.315
Ingredient 7 (*x*_7_)	−0.855	−0.915	−0.944	−0.967	−0.906	−0.949	−0.977	−0.923	−0.935	−0.935	−0.902	−0.877	−0.990	−0.946
Ingredient 8 (*x*_8_)	0.220	−0.253	−0.523	−0.500	−0.045	−0.672	−0.730	−0.178	−0.668	−0.700	−0.353	1.000	−0.938	0.608
Ingredient 9 (*x*_9_)	0.131	0.434	−0.232	0.616	0.051	−0.596	−0.232	−0.010	0.030	0.697	−0.717	0.354	−0.434	−0.434
Ingredient 10 (*x*_10_)	−0.398	−0.456	−0.772	−0.563	−0.645	−0.686	−0.918	−0.247	0.398	−0.415	0.944	0.301	−0.538	−0.643
Ingredient 11 (*x*_11_)	−0.836	0.127	−0.154	0.511	−0.310	−0.081	−0.673	−0.550	−0.789	0.615	−0.629	−0.456	0.335	0.563
Ingredient 12 (*x*_12_)	0.083	−0.436	−0.635	−0.591	−0.127	−0.779	−0.919	−0.193	−0.702	−0.646	−0.425	1.000	−1.000	0.304
Ingredient 13 (*x*_13_)	−0.350	1.000	0.898	0.721	0.196	−0.713	−0.042	−0.599	0.193	−0.576	−0.042	0.112	0.089	0.460
Ingredient 14 (*x*_14_)	−0.214	−1.000	−0.002	−0.070	−1.000	0.748	−0.282	−0.186	−0.179	−1.000	−1.000	−1.000	0.421	−0.165
Ingredient 15 (*x*_15_)	−0.907	−0.928	−0.717	−0.855	−0.643	−0.236	1.000	−0.794	−0.579	−0.723	−0.858	−0.926	−0.759	−0.762
Ingredient 16 (*x*_16_)	0.010	0.010	−0.709	−0.559	−0.409	−0.949	−0.529	−0.109	−0.079	−0.529	−0.529	−0.589	−0.349	−0.679

### 3.3 Mathematical sensory model

Each sensory attribute have been expressed has a function of the chemical composition by using a RBF model of the following form:

(9) $${\hat{A}}_{i}\left(x\right)=a_{i,0}+\sum_{j=1}^{16}a_{i,j}\exp(-||x-m_{i,j}{\left|\right|}^{2}/\sigma_{i,j}^{2}),\;i=1,\cdots ,8$$

**Figure 3:**
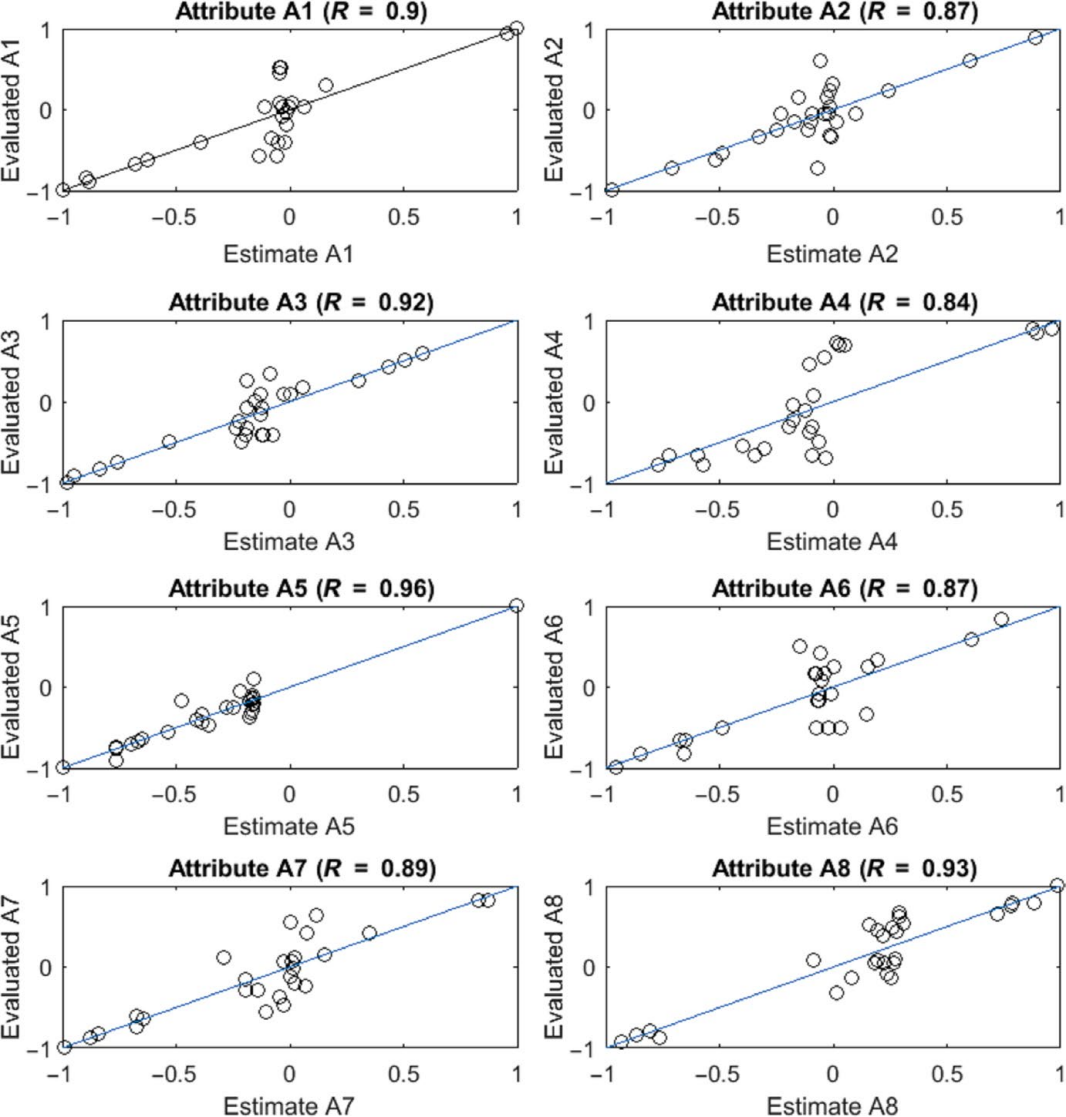
Goodness of fit of the sensory model

The parameters of this model have been tuned by using the Neural Network Toolbox (Beal et al., 2018), over the database given in Tables 2, 3, 4, and 5. The Figure 3, shows the goodness of fit of each sensory attribute. We can see that the coefficient of determination R is very good for each attribute, i.e., at least 84% of the total variation is explained by the sensory model.

### 3.4 Sensory specifications and optimization

The sensory specifications must be given in accordance with the hedonic score of each product. This can be done by determining which sensory attributes contributes to a high hedonic score. To this end, a linear regression over the normalized sensory attributes has been performed with the following model

(10) $$\hat{h}=\alpha_{0}+\sum_{i=1}^{8}\alpha_{i}A_{i}$$

The following parameter vector has been found via a classical least square regression.

(11) α=[5.77,0.007,0.331,-0.268,-0.114,0.0279,0.053,0.299,0,417]

**Figure 4:**
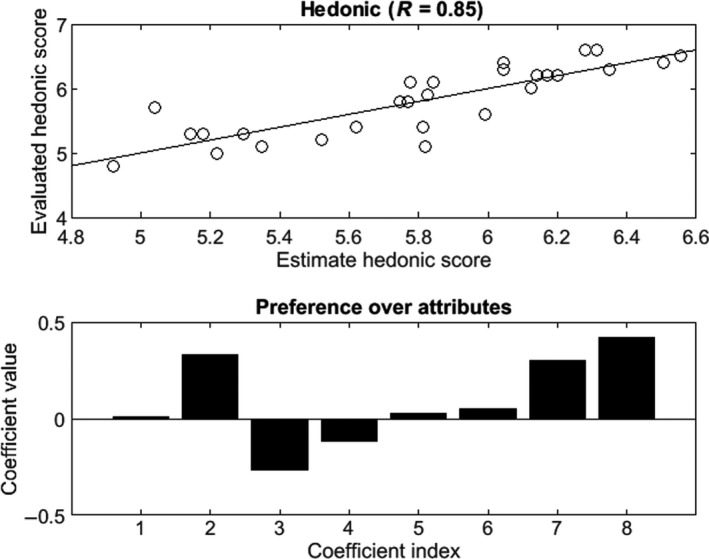
Preference order over the sensory attributes

The top of Figure 4 shows the goodness of fit of this linear regression with a quite good coefficient of determination. The bottom of Figure 4 shows the value of the coefficient related to each sensory attribute. We can see that the attribute $A_{8}$, $A_{2}$, and $A_{7}$ play an important role in the appreciation of the product with the order of importance $A_{8}>A_{2}>A_{7}$. The other attributes have a minor role or have a negative impact on the hedonic score, which is the case for the attributes $A_{4}$ and $A_{3}$, with $A_{4}<A_{3}$.

The preference order is then the following one

(12) $$\left(A_{8},\;A_{2},\;A_{7},\;A_{6},\;A_{5},\;A_{1},\;A_{4},\;A_{3}\right)$$

Consequently, to find the best composition we have to solve the following optimization problem

(13) $$\begin{array}{c} \mathrm{maximize}\;w_{8}{\hat{A}}_{8}\left(x\right)+w_{2}{\hat{A}}_{2}\left(x\right)+w_{7}{\hat{A}}_{7}\left(x\right)+\frac{1}{c(x)}\\ \mathrm{subject}\;\mathrm{to}:\;x\in X\\ \;{\hat{A}}_{2}\left(x\right)<{\hat{A}}_{8}\left(x\right)\\ \;{\hat{A}}_{7}\left(x\right)<{\hat{A}}_{2}\left(x\right)\\ \;{\hat{A}}_{6}\left(x\right)<{\hat{A}}_{7}\left(x\right)\\ \;{\hat{A}}_{5}\left(x\right)<{\hat{A}}_{6}\left(x\right)\\ \;{\hat{A}}_{1}\left(x\right)<{\hat{A}}_{5}\left(x\right)\\ \;{\hat{A}}_{4}\left(x\right)<{\hat{A}}_{1}\left(x\right)\\ \;{\hat{A}}_{3}\left(x\right)<{\hat{A}}_{4}\left(x\right) \end{array}$$

where $X={\left[-1,1\right]}^{16\times 28}$ (indeed recall that the ingredients have been normalized between [-1,1]), and c(x) is the financial cost that we want to minimize. This financial cost is assumed to be an increasing function of the product aging time. The solution of this optimization problem ensure the preference order 12 while maximizing the attributes $A_{8}$, $A_{2}$, and $A_{7}$ and minimizing the price product. The weights traduce the relative importance of the attributes to be maximized. These weights have been set in accordance with their coefficient, i.e., we have

(14) $$w_{2}=\frac{\alpha_{2}}{\alpha_{2}+\alpha_{7}+\alpha_{8}},\;w_{7}=\frac{\alpha_{7}}{\alpha_{2}+\alpha_{7}+\alpha_{8}},\;w_{8}=\frac{\alpha_{8}}{\alpha_{2}+\alpha_{7}+\alpha_{8}}$$

Solving the optimization problem 13 gives the following normalized optimal composition

(15) $$\begin{array}{c} x^{*}=\;[-1,000,\;-1,000,\;-0,296;-0,561,\;-0,097,\;-0,686,\;-0,518,\;1.000,\;0,206,\ldots \\ \;\;1.000,\;-0,469,\;-1.000,\;-0,849,\;-0,132,\;-0,356,\;-0,638] \end{array}$$

The MATLAB code used to obtain a solution to the optimization problem 13 is presented in Appendix S1.

## 4 CONCLUSIONS

In this paper, a new optimal approach has been developed to design products in accordance with the users perception. To this end, three kind of information have been exploited: the product space, the sensory analysis over the product space, and some physical quantities extracted from the product space. Sensory analysis has been used to study how a given product is perceived with respect to some sensory attributes. It is assumed that these sensory attributes are related to some physical quantities of the product. These information have then be combined to obtain a mathematical sensory model based on RBF basis functions. To this end, the theory of function approximation has been used as a general tool. This sensory model has then been used to formulate a constrained nonlinear programming problem that take into account the sensory specifications expressed as a preference order over the sensory attributes. The solution of this optimization problem gives thus the best user‐centered parameters design that have to be used for the conception of the final product. Finally, a practical example has been presented to illustrate the various steps of the proposed method and has shown its applicability.

## CONFLICT OF INTEREST

The authors declare that they do not have any conflict of interest.

## ETHICAL STATEMENT

This study is based on the data base available in Serafim et al. (2013). Consequently human and animal testing is unnecessary.

## ENDNOTE

1 Note that any maximization problem can be converted into a minimization problem. Indeed maximizing f(x) is the same as minimizing -f(x).
