# Supplementary material for: Toward an optimal quantitative design method integrating user‐centered qualitative attributes
Source: Food Sci Nutr. 2019 May 29;7(7):2261–70. doi: 10.1002/fsn3.1058 (PMC6657721; doi:10.1002/fsn3.1058)
Supplement: Supplementary file 1 [file FSN3-7-2261-s001.pdf]

Appendices

A MatLab code

The MatLab code used to obtain a solution to the optimization problem (**??**) related to the practical example presented in section **??** is given here after.

```
function sol_opt=case_study(X,Y,month)

% X, Y, month are the CachaÃ§as data presented in the following paper
%
% Felipe A. T. Serafim, Fernanda R. F. Seixas, Alexandre A. Da Silva,
% Carlos A. Galinaro, Eduardo S. P. Nascimento, Silmara F. Buchviser.
% Luigi Odellob and Douglas W. Franco.
% Correlation between Chemical Composition and Sensory Properties
% of Brazilian Sugarcane Spirits (CachaÃ§as). Journal of the Brazilian
% Chemical Society, Vol. 24(6), pp. 973-982, 2013.

% Sensory model
RBF1=newrb(X,Y(1,:),0,1,10);
RBF2=newrb(X,Y(2,:),0,1,10);
RBF3=newrb(X,Y(3,:),0,1,10);
RBF4=newrb(X,Y(4,:),0,1,10);
RBF5=newrb(X,Y(5,:),0,1,10);
RBF6=newrb(X,Y(6,:),0,1,10);
RBF7=newrb(X,Y(7,:),0,1,10);
RBF8=newrb(X,Y(8,:),0,1,10);

% Financial cost related to the aging time
p=120*(1-exp(-month/15));
RBFp=newrb(X,p',0,1,10);

% Objective function
func=@(x)     -0.3154*RBF2(x)-0.2854*RBF7(x)-0.3991*RBF8(x)+RBFp(x);

% Optimization
opt=optimoptions('fmincon');
opt.Display='iter-detailed';%'none';

x0=(max(X')'-min(X')').*rand(16,1)+min(X')';
sol_opt = fmincon(func,x0,[],[],[],[],min(X')',max(X')',...
          @(x) mycon(x,RBF1,RBF2,RBF3,RBF4,RBF5,RBF6,RBF7,RBF8),opt);
end

function [cin,ceq] = mycon(x,RBF1,RBF2,RBF3,RBF4,RBF5,RBF6,RBF7,RBF8)
% Preference order constraints
          ceq = [];
          cin=[RBF2(x)-RBF8(x)+0.01;...
               RBF7(x)-RBF2(x)+0.01;...
               RBF6(x)-RBF7(x)+0.01;...
```

```
                    RBF5(x)-RBF6(x)+0.01;...
                    RBF1(x)-RBF5(x)+0.01;...
                    RBF4(x)-RBF1(x)+0.01;...
                    RBF3(x)-RBF4(x)+0.01];
end
```
